# Lessons Learned from an Attempted Pragmatic Randomized Controlled Trial for Improvement of Chronic Pain-Associated Disability in Green Professions: Long-Term Effectiveness of a Guided Online-Based Acceptance and Commitment Therapy  (PACT-A)

**DOI:** 10.3390/ijerph192113858

**Published:** 2022-10-25

**Authors:** Lina Braun, Yannik Terhorst, Ingrid Titzler, Johanna Freund, Janika Thielecke, David Daniel Ebert, Harald Baumeister

**Affiliations:** 1 Department of Clinical Psychology and Psychotherapy, Institute of Psychology and Education, University of Ulm, 89081 Ulm, Germany; 2 Department of Research Methods, Institute of Psychology and Education, University of Ulm, 89081 Ulm, Germany; 3 Department of Clinical Psychology and Psychotherapy, Institute of Psychology, Friedrich-Alexander University of Erlangen-Nürnberg, 91052 Erlangen, Germany; 4 Faculty TUM Department of Sport and Health Sciences, TU Munich, 80992 Munich, Germany

**Keywords:** internet intervention, acceptance and commitment therapy, ACT, chronic pain, farmers, gardeners, foresters, 6-month follow-up, 12-month follow-up, randomized controlled trial

## Abstract

Musculoskeletal symptoms are increased in farmers, whereas the prevalence of chronified pain is unknown. Online interventions based on acceptance and commitment therapy (ACT) have shown encouraging results in the general population, representing a promising approach for reducing pain interference in green professions (i.e., farmers, foresters, gardeners). We conducted a pragmatic RCT comparing a guided ACT-based online intervention to enhanced treatment-as-usual in entrepreneurs, contributing spouses, family members and pensioners in green professions with chronic pain (CPG: ≥grade II, ≥6 months). Recruitment was terminated prematurely after 2.5 years at *N* = 89 (of planned *N* = 286). Assessments were conducted at 9 weeks (T1), 6 months (T2) and 12 months (T3) post-randomization. The primary outcome was pain interference (T1). The secondary outcomes encompassed pain-, health- and intervention-related variables. No treatment effect for reduction of pain interference was found at T1 (β = −0.16, 95%CI: −0.64–0.32, *p* = 0.256). Improvements in cognitive fusion, pain acceptance, anxiety, perceived stress and quality of life were found only at T3. Intervention satisfaction as well as therapeutic and technological alliances were moderate, and uptake and adherence were low. Results are restricted by low statistical power due to recruitment issues, high study attrition and low intervention adherence, standing in contrast to previous studies. Further research is warranted regarding the use of ACT-based online interventions for chronic pain in this occupational group. Trial registration: German Clinical Trial Registration: DRKS00014619. Registered: 16 April 2018.

## 1. Introduction

Chronic pain is a burdensome chronic somatic condition leading to substantial decrease in life quality on an individual level [1,2] as well as high health care costs on a societal level [2]. Chronic pain is usually defined as persisting for at least three to six months [3]. Following ICD-11, chronic pain is classified as persistent or recurrent pain for a duration of at least three months, entailing chronic pain conditions ranging from chronic primary pain to chronic secondary musculoskeletal pain [4].

The prevalence of chronic pain operationalized as constant or frequently recurrent pain for more than three months is 28.3% in the German population [5], similar to a prevalence rate of 27% previously estimated based on studies conducted in the European Union [6]. Yet, prevalence rates can differ in population subgroups and were found to be higher in rural residents, in persons with lower educational level or in persons living in poverty, based on a US survey [7]. In addition, increased occupation-related risk for musculoskeletal disorders like back pain is reported for occupations with physically strenuous and manual labor, for instance, in the agricultural sector [8,9,10].

In line with this, increased prevalence rates of musculoskeletal symptoms were reported for farmers in comparison to non-farmers [11,12]. Overall, high levels of musculoskeletal symptoms were reported in farming populations in Europe and beyond [13,14,15], but also specifically in Germany [16,17]. Further, a variety of agricultural risk factors for serious injury were identified, such as being a full-time versus a part-time farmer or a farm owner or operator versus a family member or hired worker [18]. To prevent and reduce occupation-related musculoskeletal pain, mostly ergonomic interventions have been explored in farming populations to date [19,20,21,22], whereas psychological interventions for reduction of pain-related disability and psychological distress in already chronified pain have not been investigated thus far in this occupational group.

Catastrophizing beliefs have been shown to intensify the perceived pain intensity, pain-related disability and psychological distress [23,24], thus being an important mechanism of change in psychological therapies [25]. Further, pain acceptance has been identified as a mediator in cognitive behavioral therapy (CBT) for chronic pain [26,27]. Thus, Acceptance and Commitment-based Therapy (ACT) specifically focusing on pain acceptance has been investigated as a psychological intervention to overcome negative beliefs and affectivity accompanying chronic pain [28]. Psychological flexibility has been proposed as a mediator of change, specifically in ACT, for reducing pain-associated disability [25,29].

ACT has been shown to be effective across different formats for reducing pain interference in chronic pain, with effect sizes of SMD = 0.62 [0.21;1.03] at post-treatment and SMD = 1.05 [0.55; 1.56] at follow-up (FU) as well as for reducing disability with effect sizes of SMD = 0.40 [0.01; 0.79] at post-treatment and SMD = 0.39 [0.11; 0.67] at FU, including FU periods ranging from 2 to 6 months after intervention completion [30]. A recent meta-analysis extended effectiveness in reduction of pain interference to online-based and predominantly guided delivery of ACT, reporting comparable effect sizes of SMD = −0.50 [−0.81; −0.20] for pain-interference at post-treatment and SMD = −0.69 [−1.14; −0.25] at FU based on five randomized controlled trials (RCTs) [31].

As online-based delivery enables anonymous and time-flexible participation from home [32], this might facilitate the uptake of such mental health care offers. According to a population-based Swedish study at the beginning of the century, farmers did not utilize health care offers more often and took less sick leave than non-farmers despite experiencing more musculoskeletal problems in different pain regions [12]. Indeed, farmers were in general only half as likely as non-farmers to consult a general practitioner or mental health professional, according to an Australian study [33]. Barriers to health care utilizations in farmers compared to non-farmers extend particularly to attitudes towards support seeking for mental health problems, such as higher levels of self-reliance and normalizing of problems [34]. Thus, a low-threshold prevention approach seems indicated in this occupational group. As this is the first study to evaluate a psychological prevention approach to reduce pain interference due to chronic pain in green professions, the extent of acceptance and utilization to be expected in this target group is unclear.

This RCT was part of the nationwide model project “With us in balance” conducted by a German social health care insurance provider for agriculture, forestry and horticulture, aiming to evaluate and implement internet- and tele-based interventions to enhance mental health and prevent depression in green professions [35,36,37,38,39,40].

In this trial, we evaluate a guided ACT-based online intervention for reduction of pain interference in persons in green professions (i.e., farmers, foresters and gardeners) and burdened by chronic pain [41]. We aim to evaluate if this intervention is effective in (a) reducing pain interference, (b) improving pain-associated and mental health-related outcomes, and (c) preventing onset or facilitating remission of potential depression in the corresponding subgroups over a FU period of 12 months. Further, as the recruitment goal for achieving confirmatory study results was missed, we placed greater emphasis on whether the intervention is (d) feasible in this pragmatic health care setting, i.e., if the intervention is satisfactory, accepted, used and safe for use in the occupational group of green professions burdened with chronic pain.

## 2. Materials and Methods

### 2.1. Study Design

In this two-armed pragmatic RCT, an ACT-based online intervention is compared to a control group (CG) receiving unrestricted access to enhanced treatment as usual (TAU+) with parallel group design. Outcome measurements at 9-week post-randomization (T1), 6-month (T2) and 12-month (T3) FUs are reported. A 24-month (T4) FU was not reported due to limited statistical power following high study attrition. A 36-month (T5) FU was dropped due to termination of recruitment before the recruitment goal was reached. This study was approved by the Ethics Committee of the University of Ulm (no. 453/17, 22 February 2018) and registered in the German Clinical Trial Registry (DRKS00014619, 16 April 2018) prior to study start. Results are reported in accordance with the CONSORT 2010 statement [42,43], the extension for reporting of pragmatic trials [44] and guidelines for internet intervention research [45]. More details are available in the corresponding study protocol [41]. Deviations from the study protocol are reported in Appendix A.

### 2.2. Inclusion and Exclusion Criteria

Those eligible for study inclusion were (a) policyholders from a social insurance company for agriculturists, foresters and horticulturists (SVLFG) in Germany working as entrepreneurs, contributing spouses, family members or pensioners in the green sector. The presence of chronic pain symptomology was operationalized according to the recommendations of the International Association of Pain, with participants being required to report (b) a pain duration of at least 6 months [3] and (c) a considerable pain intensity of at least grade II according to the Chronic Pain Grade questionnaire (CPG) [46]. Further eligibility criteria were (d) age ≥ 18, (e) having an email address, (f) having internet access and (g) willingness to give informed consent. Exclusion criteria were (a) ongoing psychotherapy, (b) inability to distance from suicidal ideation by signing a non-suicide contract (if suicide item > 1 of the Beck Depression Inventory (BDI-II) [47]) due to ethical reasons to ensure patient safety (in this case, patients underwent a standardized safety procedure described in the study protocol [41] and were, if necessary, referred into adequate treatment in routine care) and (c) eligibility and preference to participate in the parallel clinical trial PROD-A [37].

### 2.3. Recruitment

Participants were recruited nationwide in Germany via common recruitment approaches of the social insurance company, entailing the dispatch of 80,000 invitation letters to policyholders, several short articles in the member journal of SVLFG (circulation 1.3 million) as well as recruitment through the SVLFG newsletter and information postings on different websites starting in February 2018. We used a pragmatic recruitment strategy that focused on recruitment for both parallel RCTs, PROD-A and PACT-A, simultaneously, meaning that recruitment measures often did not specifically address chronic pain symptomology. Recruitment measures addressed universally und non-selectively potentially interested policyholders. The realization of recruiting measures (i.e., dispatch of invitation letters) specifically addressing policyholders with a medical history of chronic pain symptomology failed due to adverse circumstances (i.e., inter alia, COVID-19 pandemic). Recruitment had to be terminated in July 2020 due to time and resource constraints, with a final sample size of *N* = 89, before the initial recruitment goal (of *N* = 286) was reached.

### 2.4. Procedure

**Figure 1:**
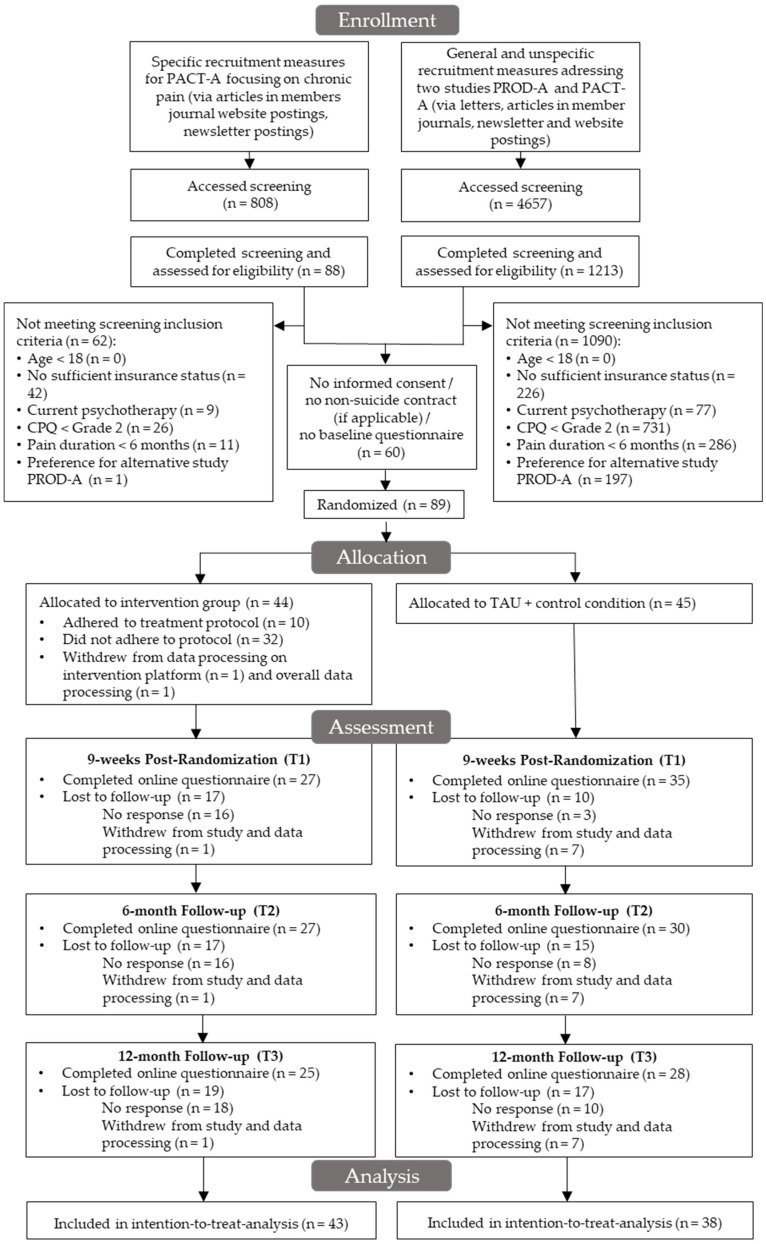
Study flow.

All recruitment measures entailed that the participants received an online link, giving access to an initial online screening to check eligibility criteria for study participation. In the case of eligibility, participants received a study invitation per email, entailing study information and informed consent. After returning the signed consent, participants were invited to the baseline survey. Upon completion of this questionnaire, participants were included into the study by randomization. Permuted block randomization on an individual level was conducted based on an automated web-based program (https://sealedenvelope.com/, accessed on 30 April 2018) with randomly arranged block sizes (2, 4, 6) and an allocation ratio to parallel study arms of 1:1. Randomization and allocation to study conditions was performed by a third party not otherwise involved in and blinded towards study conduct. Data collectors were blinded towards group membership of participants. Due to the study design, participants themselves could not be blinded towards group membership. Participants were invited regularly to FU online assessments after randomization. The study flow is presented in Figure 1.

### 2.5. Intervention

Participants of both study conditions had unrestricted access to treatment as usual (TAU). The actual use of routine health care services was monitored with a version of the Trimbos Institute and Institute of Medical Technology Questionnaire for Costs Associated with Psychiatric Illness (TiC-P) [48], adapted to the occupational context of green professions to determine potential differences between study groups.

#### 2.5.1. Control Group

The CG received a short 5-page psychoeducation material per email, containing information about (1) stress and risk factors for mental distress and disorders, (2) depression and chronic pain as well as indicated prevention, treatment and relapse prevention offers, (3) self-help offers and self-care tips for policyholders, (4) inpatient and outpatient psychotherapeutic options in routine care, and (5) emergency numbers and contact options in the case of acute crises. Thus, TAU was enhanced to TAU+ by enclosure of the information material.

#### 2.5.2. Intervention Group

The intervention group (IG) received access to an eCoach guided online intervention named GET.ON Chronic Pain that was based on the principles of ACT. The training was multimodal and interactively designed, combining picture, audio and video material with psycho-educative information, exercises and exemplary personas. The online intervention was based on the previously evaluated online intervention ACTonPain for chronic pain patients [49,50] and adapted to the green sector in regard to the audiovisual content. The training consisted of 7 modules (30–60 min each) focusing on mindfulness exercises (1), acceptance (2), negative thoughts and emotions (3), reflection on self-concept (4), values (5), commitment to goals (6) and future planning (7). The online intervention was unlocked after an initial contact between the participant and assigned eCoach. During the training phase, participants received feedback on each completed module from their eCoach. After the training phase, participants were contacted monthly during a consolidating phase of 12 months by their eCoach. Contacts between participants and eCoaches took place either via telephone or in written form (via internal messaging system) depending on the preference of participants. eCoaches from the GET.ON institute were psychologists, psychotherapists in training or licensed psychotherapists and received supervision from a licensed psychotherapist. Upon request of the participant and indication present, participants were able to receive a second guided online intervention after completion of GET.ON Chronic pain if the eCoach and the supervising licensed psychotherapist agreed. As a second online intervention, a portfolio regarding depressive symptoms with (GET.ON Mood Enhancer Diabetes [51]) and without (GET.ON Mood Enhancer [52]) comorbid diabetes, chronic stress (GET.ON Stress [53]), insomnia (GET.ON Recovery [54]), harmful alcohol consumption (GET.ON be clever—drink less [55]), as well as panic and agoraphobia (GET.ON Panic [56]) was available for selection as described in the study protocol of the parallel trial PROD-A [37]. More details on the intervention protocol can be found in the corresponding study protocol of PACT-A [41].

### 2.6. Outcome Measures

#### 2.6.1. Primary Outcome

Pain interference was assessed as primary outcome at 9 weeks post-randomization (T1) with a subscale of the Multidimensional Pain Inventory (MPI) [57]. The pain interference subscale of the MPI consists of 10 items rated on a 7-point scale ranging between 0 and 60 points, evaluating the extent of pain interference in different areas of life. Reliability is reported to be high, with α = 0.94 [58].

#### 2.6.2. Pain-Related Secondary Outcomes

Pain interference was also recorded at T2 and T3 with the MPI subscale. The Brief Pain Inventory (BPI) [59,60] was additionally applied as a 7-item scale rated from 0 to 10 to assess the extent of pain-related interference with function regarding different life areas in the last 24 h. A high reliability of α = 0.88 is reported for this scale [59]. Pain intensity regarding worst, least, average and current pain experienced in the last week was evaluated with a 11-point numerical scale from 0–10. Perceived improvement was assessed with a single item rated on a 7-point scale at each measurement point as a measure of global improvement following treatment in a clinical trial based on the Patient Global Impression of Change scale (PGIC) [61]. In addition, ACT-based measures were applied, including the 20-item Chronic Pain Acceptance Questionnaire (CPAQ), with good reliability of total and subscales of between α = 0.84–0.87 [62]; the 7-item Cognitive Fusion Questionnaire (CFQ), characterized by excellent reliability (α = 0.94) [63]; as well as the 18-item Committed Action Questionnaire (CAQ), with a high reliability of α = 0.91 [64] to assess different aspects of the extent of psychological flexibility in chronic pain management.

#### 2.6.3. Mental Health–Related Secondary Outcomes

Depressive symptom severity was assessed with the 16-item version of the Quick Inventory of Depressive Symptomology (QIDS-SR16). The scale is characterized by high reliability (α = 0.86) [65]. QIDS-SR16 and a self-report version of the Composite International Diagnosis Interview (CIDI-SC) in the WHO World Mental Health Surveys International College Student Project version [66] were both applied to assess onset and remission of potential depression. The 7-item Generalized Anxiety Disorder questionnaire (GAD-7) with a high internal consistency of α = 0.89 [67] was used to evaluate generalized anxiety symptoms. The extent of stress load was evaluated with the 10-item version of the Perceived Stress Scale (PSS), having acceptable internal consistency (α = 0.78) [68]. The severity of insomnia symptoms was assessed with the Insomnia Severity Index (ISI), reported to have good reliability (α = 0.81) [69]. Harmful alcohol consumption was evaluated with the Alcohol Use Disorder Identifications Test (AUDIT) [70,71], with satisfactory reliability for the subscales ranging between α = 0.80 and α = 0.83 [72,73]. The Assessment of Quality of Life (AQoL-8D), reported to have excellent reliability (α = 0.96) [74], was applied for evaluation of quality of life.

The subjective prognosis of employment scale (SPE) was applied to evaluate if participants expected to be able to work until retirement. A value of rep = 0.99 was reported for this almost perfect Guttman Scale [75].

#### 2.6.4. Intervention-Related Secondary Outcomes

The Working Alliance Inventory was applied to assess therapeutic alliance between eCoach and participant from participant (WAI-SR) and eCoach (WAI-SRT) perspectives. WAI-SR has very high reliability (α = 0.90–0.93) [76]. For assessing the eCoach perspective, a 10-item therapist version of the WAI-SRT was used (developed by Adam O Horvath, http://wai.profhorvath.com/, accessed on 19 December 2017). The reliability of a later-published 12-item version was α = 0.94 [77]. Similarly, a working alliance between online intervention and participant was assessed with the Working Alliance Inventory for Online Interventions—Short Form (WAI-TECH-SF, formerly referred to as the Technological Alliance Inventory (TAI-OT)), reported to have excellent reliability (α = 0.97) [78]. Results of the five-point Likert scale of the WAI-SR were interpreted according to a previously suggested classification of the mean item scores when dividing the sum scores by the number of items (low: 1.00–2.44, medium: 2.45–3.44, high: 3.45–5.00) [79]. As we further used WAI-SRT and WAI-TECH-SF based on seven-point Likert scales, we used an analogue classification of the mean item scores (low: 1.00–2.99, medium: 3.00–4.99, high: 5.00–7.00). Furthermore, item batteries were formulated by the authors to evaluate intervention content and record problems with intervention conduct and reasons for not starting or for discontinuing the intervention. The Client Satisfaction Questionnaire adapted to Internet interventions (CSQ-I) [80] was applied to assess user satisfaction, as an instrument attaining very good reliability (ω = 0.93–0.95). The negative effects of psychotherapy were evaluated with a version of the Inventory for the Assessment of Negative Effects of Psychotherapy (INEP) [81], adapted for online interventions.

### 2.7. Data Analysis

Data analysis was based on the Intention-To-Treat (ITT) principle. All analyses except for the primary outcome were based on two-sided tests. *p* ≤ 0.05 was defined as the significance level. Data analysis was conducted with R [82].

An a priori power analysis [41] estimated the targeted sample size to be *N* = 286 based on an expected treatment effect of d = 0.42 [83] at an alpha level of 5% of a one-tailed t-test, a power of 90% and an expected dropout rate of 31% [84]. Power analyses were conducted with G Power (3.1.9.2 and 3.1.9.4, accessed from Heinrich-Heine-University Düsseldorf, Düsseldorf, Germany).

Multiple imputation by chained equations (R package “mice”) [85] was applied based on the assumption that data is missing at random [86]. Due to the high attrition rate of up to 40% and above, 40 data sets were imputed, aiming to reduce the power fallout to less than 1% in accordance with a recommendation based on Monte Carlo simulations [87]. Imputation was performed based on predictive mean matching [88] and with the number of iterations set to *n* = 15. ITT analysis was performed with all imputed data sets. Parameter estimates were pooled in line with Rubin’s Rules [89,90].

Linear regression models were calculated for continuous variables. Standardized regression estimates (β) are reported along with 95% confidence intervals (CIs) based on robust standard errors. Linear regression models were adjusted for baseline values except for the primary outcome. Cohens’ d was estimated as the effect size. Reliable change index [91] was calculated assuming a reliability estimate of α = 0.94 for the subscale pain interference of the MPI [58]. A standard deviation unit of change of z = 1.96 (equaling *p* = 0.05) was used as a conservative criterion for both reliable improvement and deterioration [92]. Odds Ratios (OR) were reported with 95% CIs based on logistic regression models.

Onset and remission of potential Major Depressive Disorder (MDD) at the respective measurement points was estimated with Poisson regression analysis, based on both categorical evaluation of QIDS-SR16 with a score ≥ 13 classifying as potential MDD [93] and a self-report version of the CIDI. Onset of MDD was evaluated in the subsample of participants not classified with a potential MDD at baseline, whereas remission of MDD was assessed in those categorized with a potential MDD at baseline. Incidence rate ratios (IRR) were reported with 95% CIs based on Poisson regression models.

Additionally, a complete-case analysis was conducted as a sensitivity analysis on observed data to corroborate the assumption of data missing at random [94].

## 3. Results

### 3.1. Participants

In the final study sample, *N* = 89 participants were included. The intervention sample was reduced by 38.6% at T1 and T2 and 43.2% at T3. The CG experienced slightly less study dropout, with 22.2% at T1, 33.3% at T2 and 37.8% at T3. The group differences in study attrition rates were statistically non-significant. Overall, one (2.3%) participant in IG and seven (15.6%) participants in CG withdrew their consent to data processing, resulting in a final data analysis sample of *n* = 43 in IG and *n* = 38 in CG. The study sample was mostly female (*n* = 57, 70.4%), typically married (*n* = 74, 91.4%) or in a partnership (*n* = 4, 4.9%), with a predominantly low educational level (*n* = 53, 65.4%) and an average age of M = 56.98 (SD = 8.65). A detailed overview of sociodemographic characteristics is provided in Table 1. Additionally, a comparison of sociodemographic characteristics between intervention completers and non-completers is included in Table S1.

### 3.2. Primary Outcome

Pain interference was measured with MPI as primary outcome at 9 weeks post-randomization (T1). No significant effect of reduction of pain interference in IG (M = 29.75, SD = 14.94) compared to CG (M = 31.81, SD = 10.56) was observed at T1 based on ITT analysis (β = −0.16, 95% CI: −0.64 to 0.32, *p* = 0.256) corresponding to an effect size of d = −0.16 [−0.59; 0.28] as shown in Table 2. Complete-case analysis yielded similar results (Table S2).

### 3.3. Pain-Related Secondary Outcomes

Similarly, no significant improvement of pain interference measured with MPI was observed in favor of IG at T2 and T3. In addition, no effect of reliable improvement on an individual level was observed at either measurement point based on MPI in ITT analysis. Only in complete-case analysis a significant effect of reliable improvement in IG compared to CG was observed at T3 (OR = 3.20, 95% CI: 1.06 to 10.23, *p* = 0.043). Further, ITT analysis revealed no significant treatment effects for other pain-related outcomes at T1 and T2.

Only at T3, a significantly more pronounced reduction of cognitive fusion (β = −0.46, 95% CI: −0.92 to −0.001, *p* = 0.050) with an effect size of d = −0.23 [−0.67; 0.20] along with a significant improvement of pain acceptance (β = 0.44, 95% CI: 0.04 to 0.84, *p* = 0.034) and an effect size of d = 0.41 [−0.04; 0.84] was observed in IG compared to CG. In complete-case analysis, similar effects on ACT-based variables were found in favor of IG. Detailed information is summarized in Table 2 and Table S2.

### 3.4. Mental Health–Related Secondary Outcomes

In ITT analysis, no significant differences were observed for mental health–related outcomes at T1 and T2. At T3, significant improvements were found regarding general anxiety (β = −0.50, 95% CI: −0.95 to −0.04, *p* = 0.033) with d = −0.32 [−0.76; 0.12], perceived stress (β = −0.49, 95% CI: −0.95 to −0.03, *p* = 0.037) with d = −0.46 [−0.90; −0.02] and quality of life (β = 0.54, 95% CI: 0.19 to 0.89, *p* = 0.003) with d = 0.63 [0.18; 1.08] in favor of IG. Comparable effects were found in the complete-case analysis. No significant group differences regarding onset and remission of depression were observed based on categorical analysis of QIDS-SR16 and on CIDI-SC in both ITT and complete-case analysis. Further details are provided in Table 2 and Table S2. The comparison of health care service use in both study groups yielded no significant differences, as seen in Table 3.

### 3.5. Intervention-Related Secondary Outcomes

Working alliance (WAI-SR) was rated as medium from a patient perspective (mean scores: T1: M = 2.95, SD = 1.36; T3: M = 2.97, SD = 1.44). From an e-Coach perspective, therapeutic alliance (WAI-SRT) was assessed to be slightly better but still fell within a medium rating (T1: M = 3.69, SD = 0.87; T3: M = 3.58, SD = 0.82). The working alliance between participants and the online program (WAI-TECH-SF) was rated to be medium at T1 (M = 4.04, SD = 2.01) and improved moderately towards T2 (M = 4.66, SD = 1.45). Overall, satisfaction with the intervention was only moderate (M = 19.98, SD = 7.30), and satisfaction with the information material in CG was distinctly lower (M = 14.16, SD = 5.77) in comparison.

### 3.6. Use of and Adherence to the Online Intervention(s)

**Figure 2:**
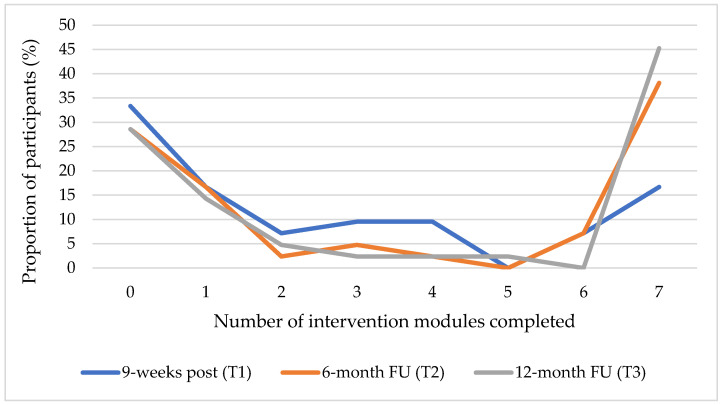
Each curve shows the proportion of participants in IG (N = 42) against the number of completed intervention modules in GET.ON Chronic Pain per measurement point (T1–T3).

Overall, *n* = 10 (of *n* = 42) participants followed treatment protocol and completed at least 6 out of 7 modules of the online intervention until 9 weeks post-randomization (T1), resulting in a total adherence rate of 23.8%. This rate increased to 45.2% at 6-month (T2) and 12-month (T3) FU, with *n* = 19 completing at least 80% of the online intervention. On average, participants completed M = 2.6 (SD = 2.7) of seven modules until T1, M = 3.5 (SD = 3.2) until T2, and M = 3.7 (SD = 3.2) modules until T3. It should be noted that 33.3% (*n* = 14) of the participants did not complete one single intervention module until T1. At T2 and T3, this rate decreased slightly to 28.6% (*n* = 12), as shown in Figure 2. Two participants were allocated at 6-month FU and further two at 12-month FU to one of the following additional guided online interventions: GET.ON Stress (*n* = 2), GET.ON Mood Enhancer (*n* = 1) and GET.ON Recovery (*n* = 1). Reasons reported for not starting the intervention, problems encountered with the intervention and reasons for intervention dropout are provided in Table S3.

### 3.7. Negative Effects and Reliable Deterioration

A total of 14 (of 27, 51.9%) participants of IG reported at least one side effect due to the online intervention at T1. This applied to 11 participants at T2 (of 27, 40.7%) and T3 (of 25, 44.0%). Overall, 31 negative effects at T1, 19 at T2 and 28 at T3 were reported in relation to the online intervention. The most often assented side effects were the neglect of hobbies and social contacts but also the feeling of being forced to do exercises and the increase of arguments in relationships. Further, no significant group differences in symptom deterioration based on ITT- or complete-case analysis of the primary outcome measurement scale for pain interference (MPI) was detected at any measurement point. Details are included in Table 2, Tables S2 and S4.

## 4. Discussion

### 4.1. Principal Results

This is, to the best of our knowledge, the first study evaluating the long-term effectiveness of a guided ACT-based online intervention in persons occupied in green professions with chronic pain regarding the reduction of pain interference. As a main result, pain interference was not reduced at any measurement point by the use of an ACT-based intervention compared to TAU+. At an individual level, the odds of reliable change of pain interference were not significantly increased by the intervention use across measurement points. Further, treatment effects on secondary outcomes were scarce and restricted to small to moderate improvements in cognitive fusion, chronic pain acceptance, anxiety, perceived stress and quality of life at 12-month FU. Further, satisfaction with the intervention as well as therapeutic and technological alliances were rated overall as only moderate. Recruitment of the trial was very difficult, leading to premature termination. Adherence rate was low, with only 23.8% of the IG completing at least 80% of the intervention until T1. This rate increased to 45.2% at T2 and T3. However, 28.6% of the IG did not complete a single intervention module.

### 4.2. Comparison to Prior Work

Regarding the null effect for the primary outcome, these results stand in stark contrast to a study evaluating a previous version of this guided ACT-based chronic pain internet intervention in a general population sample, reporting moderate to large treatment effects regarding reduction of pain interference after 9 weeks and 6 months post-randomization compared to a waitlist CG [50]. Likewise, a recent meta-analysis pooled the few available studies to date for guided ACT-based chronic pain online interventions compared to non-ACT interventions, attaining a moderate effect of SMD = −0.50 post-treatment and a large effect of SMD = −0.69 at 6-month FU regarding reduction of pain interference [31]. Broadening the focus to include online interventions for chronic pain or functional somatic syndrome that were mostly guided and predominantly based on CBT or third-wave approaches like ACT, smaller average treatment effects of SMD = −0.35 and SMD = −0.18 emerged for reduction of functional interference at post and 6- or more months FU, respectively, compared to passive controls and SMD = −0.15 and SMD = −0.20 emerged at post and 6- or more months FU, respectively, compared to active controls [97]. In terms of secondary outcomes, we observed some delayed exploratory treatment effects after 12 months post-randomization, indicating an intervention effect of cognitive defusion and increased pain acceptance along with an improvement in anxiety, perceived stress and quality of life. This is in line with the previously suggested possibility of an incubation effect based on a meta-analytic review on online-based ACT interventions for chronic pain describing a strengthening of treatment effects over time [31]. However, the increase in completed modules over time might also have contributed to a delay of treatment effects.

Several reasons are conceivable for our findings. First and foremost, our null finding regarding the primary outcome has to be considered against the backdrop of highly limited statistical power, as the recruitment had to be terminated preliminary at approximately 31% of the targeted sample size. Post hoc power analysis with the estimated effect size of d = 0.16, alpha level of 5% for a one-tailed t-test and sample sizes *n*_1_ = 43 und *n*_2_ = 38 revealed a statistical power of only 18%. Thus, the probability of Type II error to falsely reject the null hypotheses lies at 82%. Thus, no reliable statement regarding effectiveness can be derived based on this attempted pragmatic RCT. Second, descriptive data show a within-group reduction in pain interference in both treatment groups. However, the ACT-based intervention did not reduce pain interference significantly beyond the effect of TAU enhanced with information material and spontaneous remission. The descriptive results indicate that, possibly, in this implementation context the offer of an ACT-based intervention for chronic pain might have no incremental added value over TAU enhanced with information material. This would be in line with previous research by Baumeister et al. [98] suggesting that an online intervention for depression treatment in patients with comorbid chronic back pain and a depressive disorder might not achieve an incremental added treatment effect following an orthopedic rehabilitation stay, as patients might have already profited substantially from unspecific routine care offers in rehabilitation. Thus, more research should be conducted to determine in which implementation contexts (e.g., prevention, treatment or aftercare with different intensity levels) the introduction of online interventions for chronic pain can incrementally improve pain interference beyond the effect of routine care. Yet again, as comparison against a minimally active CG like TAU+ provides a stronger statement regarding usefulness of the intervention in routine care than comparison with a waitlist CG, sufficient statistical power is even more important in order to make a comparison with TAU [99]. In line with this, a previous RCT evaluating a former version of this ACT-based online intervention in the general population observed a significant treatment effect in reduction of pain interference, but in comparison against a waitlist CG [50]. Third, adherence was exceptionally low at 9 weeks post-randomization and increased over time. Similar to a parallel trial evaluating a tailored online intervention program for persons occupied in green professions and at risk for depression [39], we observed a pragmatic intervention use over a large time frame of several months. This is in line with participants reporting time restrictions due to high workload in work and private life as key barriers to intervention use in the context of qualitative interviews conducted [100]. Thus, it seems the primary outcome was terminated too early at 9 weeks after randomization. As intervention adherence is associated with outcomes of psychological internet interventions [101], treatment dose might have been too low overall to achieve a reduction of pain interference over the effect in the enhanced TAU group after 9 weeks. However, not even a delayed between-group treatment effect on pain interference emerged over the time frame of 12 months after randomization, even though adherence to the intervention increased up until the 6-month follow up.

Fourth, pain interference and intensity at baseline were somewhat lower than in a comparable trial [50]. Possibly, severity of pain interference or pain intensity at baseline could be a potential moderator of the treatment effect. However, pain intensity has not been identified as a moderator for chronic pain outcomes in online primarily self-guided CBT-based [102] and ACT-based [103] interventions. Indeed, in previous research, no sociodemographic, medical or physical impairment variables could be consistently identified as predictors of outcome for conventional behavioral or cognitive behavioral treatment of chronic pain [104]. Fifth, research of non-digital (cognitive) behavioral treatment of chronic pain over the last decades has shown differential effectiveness mostly due to psychological process variables [104]. Specifically, psychological wellbeing [103] and psychological flexibility [25,29] were identified as moderators for ACT-based online therapy. Due to the absence of a treatment effect regarding pain interference, we were not able to investigate these variables as potential moderating and mediating variables. This sample can be classified regarding psychological wellbeing on average as subliminally depressed and anxious at baseline and well below the reported population norms for quality of life for this age group [105]. Possibly, the level of psychological flexibility and wellbeing in the recruited sample were too low to achieve a treatment effect. However, in a comparable pragmatic trial reporting robust treatment effects, similar baseline levels for baseline depression, anxiety and pain acceptance were reported [50]. Sixth, recent studies have shown that higher pain resilience could be associated with less pain catastrophizing in older adults with back pain [106]. Thus, a higher pain resilience in this population group compared to the general population might be a possible reason for experiencing fewer negative beliefs und lower psychological distress due to chronic pain, which would in turn explain why this population sample did not respond well to the psychological ACT-based treatment approach. However, we cannot determine the extent of pain resilience in this sample based on our data. As there is no research literature pertaining pain resilience in green professions, this is a hypothesis to be investigated in future studies. Seventh, participants included in this trial were on average somewhat older than participants included in a recent meta-analysis evaluating ACT-based online interventions for chronic pain [31]. Older adults with chronic pain have been shown to respond better to group-based face-to-face ACT than CBT [107]. However, a systematic review suggested relatively low overall eHealth literacy in older adults [108]. Further, rural regions have been associated with low levels of digital health literacy [109]. Along with a predominantly low education level, we might have recruited a sample generally low in digital health literacy, possibly resulting in a reduced willingness or ability to process health-related information online.

Eighth, treatment expectancy has been shown to be positively associated with outcomes in cognitive behavioral and physical treatment of chronic pain [110,111]. As we assume low digital health literacy [109] along with higher levels of stigma regarding mental health problems in older adults in rural regions [112], and specifically in farmers [113], a focus on primarily the somatic aspects of chronic pain in this target group is imaginable. If a primarily somatic disease and treatment model of chronic pain dominates in this occupational group, this would possibly limit treatment expectancy for a psychological treatment approach. Further, this would be in line with the high percentage of participants reporting at baseline to have consulted a primary care clinician (90.1%) or to have consumed pain medication (67.9%) compared to the percentage of participants reporting at baseline to have undergone psychological pain treatment before (1.2%). Further, this would be consistent with the low recruitment success, low adherence and high attrition rate experienced in this study sample. A primarily somatic treatment concept in patients with chronic somatic diseases neglecting mental health aspects can be further hypothesized as a possible explanation against the background of limited results regarding the use of internet interventions for the management of depression in chronic back pain [98] or in coronary heart disease [114]. Thus, measures to raise awareness of psychological treatment options in the context of chronic pain would be indicated, possibly by involving primary care clinicians, which were most frequented by the study sample compared to other health care options. Lastly, this is a pragmatic trial in a routine care setting. Whereas efficacy of internet interventions for different mental and somatic disorders is well confirmed, effectiveness trials in routine care settings often reveal challenges in the uptake, use and benefit of internet interventions, especially when they are unguided [115]. The evaluation of uptake and adherence of a previous unguided version of this ACT-based online intervention for chronic pain in a pragmatic setting without the supporting structures of an RCT study revealed low uptake and an exceptionally low intervention adherence rate [116]. However, our participants received regular guidance optionally via written message or telephone and were embedded in a structured study setting. Yet, possibly a more intensive, tailored or imminent guidance format as practiced in some other programs [117] might be required to facilitate adherence in this specific occupational group.

In line, this trial also provided information about feasibility of this ACT-based online intervention to the occupational group of green professions. Overall, the intervention generated little interest in the target group, as shown by low recruitment success, lower satisfaction ratings, higher study attrition and fewer completed modules post-treatment compared to the guided ACT-based intervention condition in a general population sample [50]. First, recruitment measures were part of a general recruitment strategy for advertising online interventions as a prevention offer for policyholders operating in green professions and thus, were most often not specifically addressed to policyholders with chronic pain history. As seen in the study flow, specific recruitment measures generated overall lower response yet had a higher success rate in addressing eligible policyholders, with 29.5% (26/88) of screening completers fulfilling screening inclusion criteria. General recruitment measures generated high response rates, yet only 10.1% (123/1213) of screening completers fulfilled screening inclusion criteria. Overall, the mix of recruitment measures applied was not feasible to develop a sufficient reach for this target population. Thus, large-scale yet simultaneously highly specific recruitment measures addressing directly policyholders with a history of chronic pain might be more suitable to achieve a high response rate in this population. Second, people in green professions might be an occupational group that is generally hard to reach and difficult to treat, due to presumed aspects like perceived stigma for mental health seeking [34,113] or limited digital or mental health literacy, with programs specifically being developed to enhance mental health knowledge [118,119]. However, the ACT-based pain intervention was rated considerably lower regarding intervention satisfaction as well as therapeutic and technological alliances, along with a slightly lower adherence rate at 12-month FU and attained lower recruitment success compared to an online intervention program for depression prevention evaluated in a parallel trial in green professions [38,39]. Therefore, in green professions, specifically, persons burdened by chronic pain did not respond well to this ACT-based intervention in comparison to persons at risk for depression receiving access to an online interventions’ portfolio for depression prevention. Third, participants of this ACT-based intervention can be mostly divided into all-or-nothing users, with most participants either not having completed one intervention module at all or having completed all intervention modules over a time frame of 6 months. Comparison of participant characteristics between intervention completers and non-completers revealed that the age difference was statistically significant, with the sample of non-completers being older by five years on average. Further, there was a trend towards a higher proportion of men and persons with a lower education level in the sample of non-completers. This is in line with research suggesting that lower digital literacy seems to be associated with higher age and lower educational level [120] as well as with the significantly higher agreement rates with technical difficulties as barriers to intervention use we observed in the context of a mixed-method study with a subsample of participants from this RCT as compared to participants of a trial evaluating a portfolio of online interventions for participants working in green professions and being at risk for depression [100]. Possibly, this subgroup of predominantly men of higher age and lower educational level burdened with chronic pain might be an especially hard-to-treat sample. Fourth, as certainly not every online intervention works for everyone, critical aspects for fit between user and intervention need to be identified. This might be different for various target groups. The fit between intervention design and user for one has not been considered in most ACT-based online interventions for chronic pain evaluated to date [121]. This might be an aspect, among others, explaining differences in adherence, acceptance and effectiveness in this occupational group compared to previous study results investigating general population samples.

### 4.3. Strengths and Limitations

Limitations of the present study encompass firstly the highly restricted statistical power due to preliminary termination of recruitment. Therefore, no reliable statement about effectiveness of the intervention can be derived based on this attempted pragmatic trial. Second, attrition rates were high, which is why we tested the robustness of results by analyzing complete cases as sensitivity analysis. Complete-case analysis yielded a few additional treatment effects but was consistent overall. Thus, ITT results seem reasonably robust despite high attrition rates. Third, due to recruitment issues, the time frame of recruitment extended over 2.5 years, with the recruited participants being exposed to different environmental influences over time (e.g., drought periods during summer time, COVID-19 crisis, etc.), thus possibly introducing bias and enhancing heterogeneity in the study sample due to different starting conditions. Fourth, representativeness of the study sample for the population working in the green sector burdened with chronic pain might be restricted, as mostly self-referred participants were included. Against the background of profound recruitment issues, it can be assumed that only highly motivated participants in regard to an online-based psychological treatment approach for chronic pain were included. Furthermore, the study sample encompassed mostly female participants, whereas men with chronic pain were presumably underrepresented compared to the general population of persons working in green professions. Beyond that, results cannot be generalized to populations with comorbid major depression, as participants with acute suicidality were excluded and referred to routine care, or to populations with comorbid cancer diagnosis, as predominantly back pain, shoulder/arm and muscle/joint pain were reported, but no participants with tumor pain were included.

We see the following strengths in the present study: first, this is the first study offering a guided online-based ACT intervention to persons working in green professions with chronic pain, extending previous promising research results to a specific target group with presumed high vulnerability for the development of chronified pain symptoms in a pragmatic healthcare setting. Second, results of this attempted pragmatic trial can be used to inform about feasibility of an ACT-based intervention in this specific target group and to generate hypotheses on how to further develop the intervention to improve feasibility. Third, we contribute to the growing research body for effectiveness of ACT-based online interventions for reduction of pain interference. The study’s results have been published to inform the research community, avoid publication bias and to stimulate further research to bridge the existing research gaps.

## 5. Conclusions

Lessons learned from this attempted pragmatic effectiveness trial revealed major issues in reaching and engaging the targeted population with the intervention in question. As a result, no treatment effect regarding reduction of pain interference was found. However, due to reduced statistical power as a consequence of recruitment issues, low intervention adherence and high study attrition, this attempted pragmatic trial is only able to provide non-confirmatory, highly exploratory results regarding the effectiveness of this intervention in the occupational group of green professions. A possible explanation for the limited treatment effects is that an ACT-based intervention might not be able to provide an added incremental value over enhanced routine care in this specific implementation context against the background of pragmatic intervention use. In addition, aspects like a possibly low level of digital literacy along with a primarily somatic disease and treatment model in the targeted occupational group could play an additional role.

Therefore, more systematic research is needed to identify predictors and moderators of ACT-based treatment in chronic pain as currently is carried out based on an individual participant data meta-analysis [122]. Further studies need to be conducted to specifically understand barriers of intervention use in persons occupied in green professions burdened with chronic pain and, consequently, to adapt online-based psychological treatment offers accordingly. If barriers could be overcome, ACT-based online interventions might pose a promising option to improve overall mental health and well-being in chronic pain patients in green professions. Finally, further research is warranted to determine and characterize implementation contexts in which online interventions can provide added value and substantially improve health care services in routine care.

## Figures and Tables

**Table 1:** Baseline characteristics per study arm.

Characteristic		Total Sample (*N* = 81)	Intervention Group (*n* = 43)	Control Group (*n* = 38)
Age, mean (SD)		56.98 ± 8.65	57.23 ± 9.50	56.68 ± 7.70
Gender	Male, *n* (%)	24 (29.6)	13 (30.2)	11 (28.9)
	Female, *n* (%)	57 (70.4)	30 (69.8)	27 (71.1)
Relationship	Single, *n* (%)	2 (2.5)	1 (2.3)	1 (2.6)
	In partnership, *n* (%)	4 (4.9)	0 (0.0)	4 (10.5)
	Married, *n* (%)	74 (91.4)	41 (95.3)	33 (86.8)
	Divorced or separated, *n* (%)	0 (0.0)	0 (0.0)	0 (0.0)
	Widowed, *n* (%)	1 (1.2)	1 (2.3)	0 (0.0)
Ethnicity	Caucasian, *n* (%)	79 (97.5)	41 (95.3)	38 (100.0)
	Other, *n* (%)	2 (2.5)	2 (4.7)	0 (0.0)
Country of birth	Germany, *n* (%)	80 (98.8)	43 (100.0)	37 (97.4)
	Other, *n* (%)	1 (1.2)	0 (0.0)	1 (2.6)
Level of education ^a^	Low, *n* (%)	53 (65.4)	28 (65.1)	25 (65.8)
	Middle, *n* (%)	15 (18.5)	9 (20.9)	6 (15.8)
	High, *n* (%)	13 (16.0)	6 (14.0)	7 (18.4)
Employment status	Entrepreneur, *n* (%)	32 (39.5)	15 (34.9)	17 (44.7)
	Contributing spouse, *n* (%)	22 (27.2)	12 (27.9)	10 (26.3)
	Contributing family member, *n* (%)	9 (11.1)	7 (16.3)	2 (5.3)
	Pensioner or spouse of pensioner, *n* (%)	14 (17.3)	9 (20.9)	5 (13.2)
	Incapacitated for work, *n* (%)	4 (4.9)	0 (0.0)	4 (10.5)
Company size (number of contributing persons) ^b^	1–4, *n* (%)	51 (76.1)	28 (77.8)	23 (74.2)
	5–9, *n* (%)	9 (13.4)	6 (16.7)	3 (9.7)
	10–24, *n* (%)	6 (9.0)	1 (2.8)	5 (16.1)
	25–49, *n* (%)	1 (1.5)	1 (2.8)	0 (0.0)
	≥50, *n* (%)	0 (0.0)	0 (0.0)	0 (0.0)
Main area of production ^b^	Dairy farming, *n* (%)	24 (35.8)	13 (36.1)	11 (35.5)
	Arable farming, *n* (%)	16 (23.9)	6 (16.7)	10 (32.3)
	Livestock farming, *n* (%)	10 (14.9)	7 (19.4)	3 (9.7)
	Fruit farming, *n* (%)	1 (1.5)	1 (2.8)	0 (0.0)
	Viniculture, *n* (%)	5 (7.5)	2 (5.6)	3 (9.7)
	Forestry, *n* (%)	0 (0.0)	0 (0.0)	0 (0.0)
	Horticulture, *n* (%)	5 (7.5)	2 (5.6)	3 (9.7)
	Biogas plant, *n* (%)	1 (1.5)	1 (2.8)	0 (0.0)
	Other, *n* (%)	5 (7.5)	4 (11.1)	1 (3.2)
Personal income per month (in Euros) ^c^	<1000, *n* (%)	8 (23.5)	3 (17.6)	5 (29.4)
	1000–2000, *n* (%)	12 (35.3)	8 (47.1)	4 (23.5)
	2000–3000, *n* (%)	0 (0.0)	0 (0.0)	0 (0.0)
	3000–4000, *n* (%)	4 (11.8)	0 (0.0)	4 (23.5)
	4000–5000, *n* (%)	1 (2.9)	1 (5.9)	0 (0.0)
	>5000, *n* (%)	0 (0.0)	0 (0.0)	0 (0.0)
	Not disclosed, *n* (%)	9 (26.5)	5 (29.4)	4 (23.5)
Minor second job ^d^	Yes, *n* (%)	6 (19.4)	4 (21.1)	2 (16.7)
	No, *n* (%)	25 (80.6)	15 (78.9)	10 (83.3)
Chronic pain region ^e,f^	Headaches, *n* (%)	14 (17.3)	9 (20.9)	5 (13.2)
	Back pain, *n* (%)	37 (45.7)	20 (46.5)	17 (44.7)
	Tumor pain, *n* (%)	0 (0.0)	0 (0.0)	0 (0.0)
	Shoulder/arm pain, *n* (%)	29 (35.8)	15 (34.9)	14 (36.8)
	Muscle/joint pain, *n* (%)	39 (48.1)	22 (51.2)	17 (44.7)
	Other, *n* (%)	17 (21.0)	8 (18.6)	9 (23.7)
Disability degree ^f^	Yes, *n* (%)	16 (30.8)	8 (29.6)	8 (32.0)
	No, *n* (%)	35 (67.3)	18 (66.7)	17 (68.0)
	Not disclosed, *n* (%)	1 (1.9)	1 (3.7)	0 (0.0)
Previous pain treatment ^f^	Yes, *n* (%)	32 (61.5)	15 (55.6)	17 (68.0)
	No, *n* (%)	20 (38.5)	12 (44.4)	8 (32.0)

^a^ Classification according to International Standard Classification of Education (ISCED), 2011 [95]: low = ISCED level 0–2, middle = ISCED level 3–4, high = ISCED level 5–8; ^b^ applied to participants currently working or employed in the green sector (*n* = 67); ^c^ applied to participants working as entrepreneurs or as spouse or family member with an employment contract (*n* = 34); ^d^ applied to participants working as contributing spouses and family members (*n* = 31); ^e^ selection of multiple options possible; ^f^ applied to participants initially reporting to suffer from physical illnesses (*n* = 52).

**Table 2:** ITT Analysis of primary and secondary outcomes at 9-week post-randomization (T1), 6-month (T2) and 12-month (T3) FU.

		IG (*n* = 43)	CG (*n* = 38)	ITT ^a^ (95% CI)	*p* ^b^	Cohen’s d (95% CI)
**Primary outcome**						
Pain interference (MPI)	Baseline	33.28 ± 12.11	35.79 ± 10.85			
	9 weeks	29.75 ± 14.94	31.81 ± 10.56	−0.16 [−0.64; 0.32]	0.256	−0.16 [−0.59; 0.28]
**Secondary outcomes**						
Pain-related outcomes						
Pain interference (MPI)	6 months	27.02 ± 14.22	28.36 ± 12.50	0.02 [−0.47; 0.50]	0.947	0.10 [−0.34; 0.54]
	12 months	26.10 ± 14.38	28.32 ± 12.01	−0.04 [−0.49; 0.42]	0.872	−0.17 [−0.60; 0.27]
Pain-related interference with function (BPI)	Baseline	30.70 ± 15.95	30.03 ± 14.02			
	9 weeks	26.30 ± 16.94	27.27 ± 15.29	−0.09 [−0.52; 0.33]	0.670	−0.06 [−0.50; 0.38]
	6 months	21.83 ± 14.56	23.85 ± 13.84	−0.17 [−0.71; 0.36]	0.520	−0.14 [−0.58; 0.30]
	12 months	21.59 ± 16.74	26.78 ± 15.78	−0.34 [−0.84; 0.16]	0.175	−0.32 [−0.76; 0.12]
Reliable change (MPI)	9 weeks	13 (30.2)	14 (36.8)	0.58 [0.22; 1.54]	0.281	-
	6 months	20 (46.5)	22 (57.9)	0.54 [0.22; 1.31]	0.175	-
	12 months	22 (51.2)	15 (39.5)	1.66 [0.64; 4.29]	0.300	-
Reliable deterioration (MPI)	9 weeks	8 (18.6)	3 (7.9)	0.88 [0.17; 4.62]	0.875	-
	6 months	5 (11.6)	5 (13.2)	0.57 [0.09; 3.60]	0.551	-
	12 months	4 (9.3)	2 (5.3)	0.88 [0.12; 6.56]	0.899	-
Pain intensity (NRS)	Baseline	17.47 ± 7.31	17.37 ± 6.49			
	9 weeks	15.48 ± 7.88	15.00 ± 6.75	0.05 [−0.39; 0.49]	0.809	0.06 [−0.37; 0.50]
	6 months	13.38 ± 7.31	14.32 ± 6.56	−0.14 [−0.69; 0.40]	0.594	−0.13 [−0.57; 0.30]
	12 months	13.66 ± 8.00	14.95 ± 7.37	−0.18 [−0.63; 0.28]	0.438	−0.17 [−0.60; 0.27]
Subjective rating of perceived improvement	9 weeks	3.63 ± 1.21	3.76 ± 1.46	−0.10 [−0.65; 0.45]	0.709	−0.10 [−0.53; 0.34]
	6 months	3.20 ± 1.48	3.71 ± 1.48	−0.34 [−0.86; 0.18]	0.193	−0.35 [−0.78; 0.09]
	12 months	3.13 ± 1.49	3.39 ± 1.38	−0.18 [−0.75; 0.38]	0.520	−0.18 [−0.62; 0.26]
ACT-based measures						
Activity engagement (CPAQ subscale)	Baseline	36.33 ± 10.22	37.37 ± 11.23			
	9 weeks	38.40 ± 13.27	37.21 ± 11.30	0.17 [−0.19; 0.53]	0.356	0.10 [−0.34; 0.53]
	6 months	40.36 ± 13.64	36.37 ± 12.79	0.37 [−0.08; 0.82]	0.102	0.30 [−0.14; 0.74]
	12 months	42.39 ± 12.44	38.93 ± 9.77	0.36 [−0.09; 0.82]	0.116	0.31 [−0.13; 0.74]
Pain willingness (CPAQ subscale)	Baseline	26.14 ± 10.01	26.24 ± 10.32			
	9 weeks	30.01 ± 10.82	29.98 ± 8.82	0.01 [−0.33; 0.35]	0.948	0.003 [−0.43; 0.44]
	6 months	31.49 ± 11.06	29.48 ± 9.36	0.20 [−0.21; 0.61]	0.320	0.20 [−0.24; 0.63]
	12 months	32.86 ± 11.17	28.49 ± 9.83	0.41 [−0.04; 0.86]	0.070	0.41 [−0.03; 0.85]
Chronic pain Acceptance (CPAQ total score)	Baseline	62.47 ± 17.77	63.61 ± 18.71			
	9 weeks	68.41 ± 21.43	67.18 ± 17.84	0.11 [−0.20; 0.43]	0.474	0.06 [−0.37; 0.50]
	6 months	71.85 ± 22.43	65.85 ± 18.85	0.33 [−0.05; 0.71]	0.085	0.29 [−0.15; 0.73]
	12 months	75.26 ± 20.70	67.43 ± 17.61	0.44 [0.04; 0.84]	**0.034**	0.41 [−0.04; 0.84]
Cognitive fusion (CFQ)	Baseline	23.35 ± 8.33	19.58 ± 9.47			
	9 weeks	19.13 ± 8.59	16.94 ± 8.16	0.02 [−0.42; 0.46]	0.926	0.26 [−0.18; 0.70]
	6 months	18.07 ± 9.13	19.05 ± 9.39	−0.34 [−0.80; 0.12]	0.145	−0.11 [−0.54; 0.33]
	12 months	17.95 ± 8.80	20.02 ± 8.80	−0.46 [−0.92; −0.001]	**0.050**	−0.23 [−0.67; 0.20]
Committed action (CAQ)	Baseline	68.74 ± 14.98	68.84 ± 16.87			
	9 weeks	71.78 ± 15.10	72.85 ± 14.56	−0.07 [−0.46; 0.32]	0.727	−0.07 [−0.51; 0.36]
	6 months	71.84 ± 16.59	70.58 ± 17.38	0.08 [−0.29; 0.45]	0.665	0.07 [−0.36; 0.51]
	12 months	74.27 ± 17.64	70.76 ± 17.48	0.20 [−0.27; 0.68]	0.387	0.20 [−0.24; 0.64]
**Mental health–related outcomes**						
Depressive symptom severity (QIDS-SR16)	Baseline	7.63 ± 3.80	7.89 ± 4.43			
	9 weeks	5.28 ± 3.40	6.41 ± 4.02	−0.26 [−0.65; 0.13]	0.187	−0.31 [−0.74; 0.13]
	6 months	5.26 ± 4.16	6.89 ± 4.60	−0.33 [−0.74; 0.09]	0.121	−0.37 [−0.81; 0.07]
	12 months	5.77 ± 4.14	6.36 ± 4.28	−0.10 [−0.54; 0.34]	0.650	−0.14 [−0.58; 0.30]
Onset of MDD (QIDS-SR16)	9 weeks	0 of 38 (0.0)	0 of 31 (0.0)	1.00 [1.00; 1.00]	NA	-
	6 months	1 of 38 (2.6)	3 of 31 (9.7)	0.95 [0.89; 1.02]	0.131	-
	12 months	1 of 38 (2.6)	1 of 31 (3.2)	0.97 [0.93; 1.02]	0.290	-
Onset of MDD (CIDI-SC)	9 weeks	1 of 35 (2.8)	0 of 26 (0.0)	1.02 [0.98; 1.07]	0.350	-
	6 months	1 of 35 (2.8)	2 of 26 (7.7)	0.97 [0.89; 1.06]	0.491	-
	12 months	2 of 35 (5.7)	2 of 26 (7.7)	0.99 [0.90; 1.09]	0.901	-
Remission of MDD (QIDS-SR16)	9 weeks	3 of 5 (60.0)	3 of 7 (42.9)	0.97 [0.89; 1.06]	0.491	-
	6 months	1 of 5 (20.0)	4 of 7 (57.1)	0.92 [0.85; 1.00]	0.062	-
	12 months	2 of 5 (40.0)	5 of 7 (71.4)	0.95 [0.86; 1.04]	0.254	-
Remission of MDD (CIDI-SC)	9 weeks	5 of 8 (62.5)	8 of 12 (66.7)	0.91 [0.77; 1.07]	0.254	-
	6 months	5 of 8 (62.5)	5 of 12 (41.7)	0.98 [0.85; 1.14]	0.837	-
	12 months	5 of 8 (62.5)	8 of 12 (66.7)	0.91 [0.77; 1.07]	0.254	-
Anxiety (GAD-7)	Baseline	7.16 ± 4.08	5.87 ± 4.08			
	9 weeks	4.38 ± 3.60	4.36 ± 3.41	−0.19 [−0.62; 0.24]	0.374	−0.01 [−0.44; 0.43]
	6 months	4.45 ± 3.75	4.77 ± 3.89	−0.26 [−0.77; 0.26]	0.318	−0.08 [−0.52; 0.35]
	12 months	3.93 ± 3.94	5.20 ± 4.06	−0.50 [−0.95; −0.04]	**0.033**	−0.32 [−0.76; 0.12]
Perceived stress (PSS)	Baseline	18.26 ± 6.60	17.76 ± 6.94			
	9 weeks	15.15 ± 8.33	14.03 ± 7.45	0.09 [−0.31; 0.50]	0.652	0.14 [−0.30; 0.58]
	6 months	14.77 ± 7.77	15.51 ± 9.05	−0.13 [−0.58; 0.33]	0.578	−0.09 [−0.52; 0.35]
	12 months	13.50 ± 7.31	16.99 ± 7.85	−0.49 [−0.95; −0.03]	**0.037**	−0.46 [−0.90; −0.02]
Insomnia (ISI)	Baseline	8.63 ± 4.91	9.08 ± 6.16			
	9 weeks	7.75 ± 5.23	7.00 ± 5.08	0.20 [−0.22; 0.62]	0.340	0.15 [−0.29; 0.58]
	6 months	7.24 ± 5.44	7.83 ± 5.69	−0.06 [−0.52; 0.41]	0.809	−0.11 [−0.54; 0.33]
	12 months	7.33 ± 6.35	7.32 ± 5.71	0.06 [−0.34; 0.46]	0.771	0.002 [−0.43; 0.44]
Alcohol consumption (AUDIT-10)	Baseline	2.58 ± 2.00	2.13 ± 1.79			
	9 weeks	2.09 ± 1.87	1.98 ± 1.74	−0.14 [−0.41; 0.13]	0.303	−0.06 [−0.50; 0.38]
	6 months	2.33 ± 1.54	1.74 ± 1.50	0.22 [−0.20; 0.63]	0.294	0.39 [−0.05; 0.83]
	12 months	1.59 ± 1.03	1.87 ± 1.39	−0.36 [−0.86; 0.13]	0.148	−0.22 [−0.66; 0.22]
Quality of life (AQoL-8D)	Baseline	69.44 ± 11.01	68.53 ± 10.26			
	9 weeks	71.09 ± 13.15	71.11 ± 11.85	−0.07 [−0.36; 0.21]	0.603	−0.002 [−0.44; 0.43]
	6 months	73.29 ± 12.83	70.12 ± 13.50	0.17 [−0.16; 0.50]	0.300	0.24 [−0.20; 0.68]
	12 months	77.14 ± 11.52	70.11 ± 10.74	0.54 [0.19; 0.89]	**0.003**	0.63 [0.18; 1.08]
Subjective prognosis of employment (SPE)	Baseline	1.51 ± 1.08	1.47 ± 0.98			
	9 weeks	1.35 ± 1.16	1.17 ± 1.05	0.14 [−0.20; 0.47]	0.425	0.16 [−0.27; 0.60]
	6 months	1.18 ± 1.01	1.19 ± 1.09	−0.04 [−0.48; 0.41]	0.874	−0.01 [−0.45; 0.42]
	12 months	1.34 ± 1.17	1.24 ± 1.11	0.07 [−0.40; 0.54]	0.780	0.09 [−0.35; 0.52]

Imputed data were used for ITT analysis. Means ± SD or *n* (%) are reported for baseline, 9-week post-randomization, 6- and 12-month FU. ^a^ We report standardized regression estimates (β) with 95% CI based on robust standard errors for between-group differences. Standardized regression estimates were adjusted for baseline, except for analysis of primary outcome. Reliable change and deterioration are reported based on OR along with 95% CI based on robust standard errors. Onset and remission of MDD are reported based on IRR along with 95% CI based on robust standard errors. ^b^ *p* values are based on two-sided testing, except for primary outcome, which is based on a one-sided test. Significant differences (*p* ≤ *0*.05) are printed in bold letters.

**Table 3:** Health care service use at 6-month (T2) and 12-month FU (T3).

	IG, No. (%)			CG, No. (%)			Difference between Groups, % (95% CI) ^a^	
Health Care Service	Baseline ^b^ (*n* = 43)	6-Month FU ^c^ (*n* = 27)	12-Month FU ^d^ (*n* = 25)	Baseline ^b^ (*n* = 38)	6-Month FU ^c^ (*n* = 30)	12-Month FU ^d^ (*n* = 28)	6-Month FU ^c^	12-Month FU ^d^
Primary care clinician	39 (90.7)	19 (70.4)	19 (76.0)	34 (89.5)	27 (90.0)	23 (82.1)	−0.20 [−0.40; 0.01]	−0.06 [−0.28; 0.16]
Psychotherapist	1 (2.3)	1 (3.7)	0 (0)	1 (2.6)	1 (3.3)	1 (3.6)	0.004 [−0.13; 0.15]	−0.04 [−0.18; 0.10]
Psychiatrist, Neurologist, Psychosomatic medicine specialist	2 (4.7)	1 (3.7)	2 (8.0)	1 (2.6)	2 (6.7)	2 (7.1)	−0.03 [−0.18; 0.12]	0.01 [−0.16; 0.19]
Pain treatment ^e^	9 (20.9)	3 (11.1)	3 (12.0)	8 (21.1)	8 (26.7)	2 (7.1)	−0.16 [−0.35; 0.05]	0.05 [−0.13; 0.24]
Psychological pain treatment ^e^	1 (2.3)	0 (0)	0 (0)	0 (0)	0 (0)	1 (3.6)	0.0 [−0.11; 0.12]	−0.04 [−0.18; 0.10]
Pain medication prescription	30 (69.8)	9 (33.3)	9 (36.0)	25 (65.8)	17 (56.7)	13 (46.4)	−0.23 [−0.45; 0.02]	−0.10 [−0.34; 0.15]
Antidepressant prescription	3 (7.0)	3 (11.1)	2 (8.0)	0 (0)	0 (0)	0 (0)	0.11 [−0.02; 0.28]	0.08 [−0.05; 0.25]

^a^ See Newcombe, 1998 [96]. ^b^ Baseline covering the previous 3 months (if not indicated otherwise) as measured with the TiC-P. ^c^ 6-month FU and ^d^ 12-month FU covering the previous 3 months (if not indicated otherwise) as measured with the TiC-P. ^e^ At time of measurement.

## Data Availability

Restrictions apply to the availability of these data. Informed consent obtained from the study participants did not include permission to make the data publicly available or to share the data with researchers outside of the project team.

## Supplementary Materials

The following supporting information can be downloaded at: https://www.mdpi.com/article/10.3390/ijerph192113858/s1, Table S1: Comparison of participants characteristics between completers and non-completers; Table S2: Results of complete-case analysis of primary and secondary outcomes at 9-weeks post-randomization (T1), 6-month (T2) and 12-month FU (T3); Table S3: Subjective ratings of the intervention, problems encountered and reasons for not starting or discontinuing the intervention at 9-weeks post-randomization (T1), 6-month (T2) and 12-month FU (T3); Table S4: Overview of negative intervention effects 9 weeks post-randomization (T1), 6-month FU (T2); and 12-month FU (T3).

## Appendix A. Deviations from Study Registration/Study Protocol

Study Registration: occurred on 16 April 2018 in German Clinical Trial Register: DRKS00014619

Study Protocol: Terhorst Y., Braun L., Titzler I., Buntrock, C., Freund, J., Thielecke, J., Ebert, D.D. and Baumeister, H. (2020). Clinical and cost-effectiveness of a guided internet-based Acceptance and Commitment Therapy to improve chronic pain-related disability in green professions (PACT-A): study protocol of a pragmatic randomised controlled trial. BMJ Open, 10:e034271. doi:10.1136/bmjopen-2019-034271

1. The 24-month (T4) and 36-month (T5) FU measurement points: We refrained from analyzing and reporting of data for the 24-month FU (T4) due to limited statistical power following high study attrition. Data collection at the initially planned measurement point after 36 months (T5) was omitted.
2. Per-protocol analysis: Due to preliminary recruitment stop, high study attrition and the low rate of intervention participants adhering to treatment protocol, no per-protocol analysis was performed. Statistical power is not sufficient to detect potential treatment effects in per-protocol analysis.
3. Complete-case analysis: Instead, an additional complete-case analysis was conducted to examine the robustness of the ITT analysis against the background of high study attrition.
4. Moderation analysis: As the study is substantially underpowered due to the reasons already stated, we have refrained from conducting a further analysis of possible moderating variables.
5. Mediation analysis: We proposed to analyze the three facets of psychological flexibility (pain acceptance, cognitive fusion and committed action) as well as technological and therapeutic alliances as mediating variables in the study protocol. As no reduction of pain interference was observed by this ACT-based online intervention, we were not able to investigate possible mediating variables of this supposed treatment effect.
6. Cost-effectiveness analysis: Due to the low statistical power, we also refrained from conducting a cost-effectiveness analysis.
