# Supplementary material for: Lessons Learned from an Attempted Pragmatic Randomized Controlled Trial for Improvement of Chronic Pain-Associated Disability in Green Professions: Long-Term Effectiveness of a Guided Online-Based Acceptance and Commitment Therapy (PACT-A)
Source: Int J Environ Res Public Health. 2022 Oct 25;19(21):13858. doi: 10.3390/ijerph192113858 (PMC9655679; doi:10.3390/ijerph192113858)
Supplement: Supplementary file 1 [file ijerph-19-13858-s001.zip › ijerph-1923794-supplementary.pdf]

International Journal of
*Environmental Research
and Public Health*

MDPI

Table S1. Comparison of participant characteristics between completers and non-completers.

**Characteristic**		**Completers (*n* = 19)**	**Non-completers (*n* = 23)**	***P* value**
Age, mean (SD)		54.79 ± 10.14	59.48 ± 8.75	<0.001
Gender	Male, *n* (%)	3 (15.8)	10 (43.5)	0.09
	Female, *n* (%)	16 (84.2)	13 (56.5)	
Relationship	Single, *n* (%)	1 (5.3)	0 (0.0)	0.71
	In partnership, *n* (%)	0 (0.0)	0 (0.0)	
	Married, *n* (%)	18 (94.7)	22 (95.7)	
	Divorced or separated, *n* (%)	0 (0.0)	0 (0.0)	
	Widowed, *n* (%)	0 (0.0)	1 (4.3)	
Ethnicity	Caucasian, *n* (%)	19 (100.0)	21 (91.3)	1.0
	Other, *n* (%)	0 (0.0)	2 (8.7)	
Country of birth	Germany, *n* (%)	19 (100.0)	23 (100.0)	n.a.
	Other, *n* (%)	0 (0.0)	0 (0.0)	
Level of education [a]	Low, *n* (%)	9 (47.4)	18 (78.3)	0.08
	Middle, *n* (%)	7 (36.8)	2 (8.7)	
	High, *n* (%)	3 (15.8)	3 (13.0)	
Employment status	Entrepreneur, *n* (%)	5 (26.3)	10 (43.5)	0.71
	Contributing spouse, *n* (%)	6 (31.6)	6 (26.1)	
	Contributing family member, *n* (%)	3 (15.8)	3 (13.0)	
	Pensioner or spouse of pensioner, *n* (%)	5 (26.3)	4 (17.4)	
	Incapacitated for work, *n* (%)	0 (0.0)	0 (0.0)	
Company size (number of contributing persons) [b]	1-4, *n* (%)	11 (73.3)	16 (80.0)	0.80
	5-9, *n* (%)	3 (20.0)	3 (15.0)	
	10-24, *n* (%)	1 (6.7)	0 (0.0)	
	25-49, *n* (%)	0 (0.0)	1 (5.0)	
	≥50, *n* (%)	0 (0.0)	0 (0.0)	
Main area of production [b]	Dairy farming, *n* (%)	3 (20.0)	9 (45.0)	0.20
	Arable farming, *n* (%)	3 (20.0)	3 (15.0)	
	Livestock farming, *n* (%)	4 (26.7)	3 (15.0)	
	Fruit farming, *n* (%)	0 (0.0)	1 (5.0)	
	Viniculture, *n* (%)	0 (0.0)	2 (10.0)	
	Forestry, *n* (%)	0 (0.0)	0 (0.0)	
	Horticulture, *n* (%)	2 (13.3)	0 (0.0)	
	Biogas plant, *n* (%)	0 (0.0)	1 (5.0)	
	Other, *n* (%)	3 (20.0)	1 (5.0)	
Personal income per month (in Euros) [c]	<1000, *n* (%)	2 (33.3)	1 (9.1)	0.78
	1000-2000, *n* (%)	3 (50.0)	5 (45.5)	
	2000-3000, *n* (%)	0 (0.0)	0 (0.0)	
	3000-4000, *n* (%)	0 (0.0)	0 (0.0)	
	4000-5000, *n* (%)	0 (0.0)	1 (9.1)	
	>5000, *n* (%)	0 (0.0)	0 (0.0)	
	Not disclosed, *n* (%)	1 (16.7)	4 (36.4)	
Minor second job [d]	Yes, *n* (%)	3 (33.3)	1 (11.1)	0.58
	No, *n* (%)	6 (66.7)	8 (88.9)	
Chronic pain region [e,f]	Headaches, *n* (%)	3 (30.0)	6 (37.5)	1.0
	Back pain, *n* (%)	7 (70.0)	12 (75.0)	1.0

	Tumor pain, *n* (%)	0 (0.0)	0 (0.0)	n.a.
	Shoulder/arm pain, *n* (%)	5 (50.0)	9 (56.3)	1.0
	Muscle/joint pain, *n* (%)	7 (70.0)	14 (87.5)	0.34
	Other, *n* (%)	4 (40.0)	4 (25.0)	0.66
Disability degree [f]	Yes, *n* (%)	3 (30.0)	5 (31.3)	1.0
	No, *n* (%)	7 (70.0)	10 (62.5)	
	Not disclosed, *n* (%)	0 (0.0)	1 (6.3)	
Previous pain treatment [f]	Yes, *n* (%)	4 (40.0)	10 (62.5)	0.42
	No, *n* (%)	6 (60.0)	6 (37.5)	

Participant characteristics were compared between participants having completed at least 80% ("completers") and having completed less than 80% ("non-completers") of the intervention modules until 12-months follow-up. *P* value refers to significance level of statistical testing for differences between completers and non-completers and is based for continuous variables on a t-test, for categorical variables on an exact Fisher's test. [a]classification according to ISCED, 2011 (OECD et al., 2015): low=ISCED level 0-2, middle=ISCED level 3-4, high=ISCED level 5-8, [b]applied to participants currently working or employed in the green sector (*n*=35), [c]applied to participants working as entrepreneurs or as spouse or family member with an employment contract (*n*=17), [d]applied to participants working as contributing spouses and family members (*n*=18), [e]selection of multiple options possible, [f]applied to participants initially reporting to suffer from physical illnesses (*n*=26).

Table S2. Complete-case-analysis of primary and secondary outcomes at 9-weeks post-randomization (T1), 6-month (T2) and 12-month FU (T3).

		**IG (n_{t0}=43, n_{t1}=27, n_{t2}=27, n_{t3}=25)**	**CG (n_{t0}=38, n_{t1}=35, n_{t2}=30, n_{t3}=28)**	**ITT[a] (95% CI)**	***P*[b]**	**Cohens d (95% CI)**
**Primary outcome**						
Pain-interference (MPI)	Baseline	33.28 ± 12.11	35.79 ± 10.85			
	9 weeks	27.67 ± 15.39	31.66 ± 10.54	-0.31 [-0.82; 0.20]	0.116	-0.31 [-0.83; 0.21]
**Secondary outcomes**						
Pain-related outcomes						
Pain-interference (MPI)	6 months	24.96 ± 15.05	27.33 ± 12.33	-0.06 [-0.50; 0.37]	0.770	-0.17 [-0.71; 0.36]
	12 months	21.28 ± 15.07	27.32 ± 12.29	-0.27 [-0.70; 0.17]	0.220	-0.44 [-1.00; 0.12]
Pain-related interference with function (BPI)	Baseline	30.70 ± 15.95	30.03 ± 14.02			
	9 weeks	23.26 ± 16.33	27.00 ± 15.23	-0.25 [-0.66; 0.15]	0.217	-0.24 [-0.75; 0.28]
	6 months	20.11 ± 15.05	23.17 ± 13.78	-0.20 [-0.62; 0.22]	0.343	-0.21 [-0.75; 0.32]
	12 months	17.92 ± 16.82	25.96 ± 15.74	-0.39 [-0.83; 0.04]	0.075	-0.49 [-1.06; 0.07]
Reliable change (MPI)	9 weeks	10 of 27 (37.0)	13 of 35 (37.1)	1.00 [0.35; 2.82]	0.993	-
	6 months	15 of 27 (55.6)	19 of 30 (63.3)	0.72 [0.25; 2.09]	0.551	-
	12 months	16 of 25 (64.0)	10 of 28 (35.7)	3.20 [1.06; 10.23]	**0.043**	-
Reliable deterioration (MPI)	9 weeks	3 of 27 (11.1)	3 of 35 (8.6)	1.33 [0.23; 7.76]	0.738	-
	6 months	2 of 27 (7.4)	3 of 30 (10.0)	0.72 [0.09; 4.69]	0.731	-
	12 months	2 of 25 (8.0)	2 of 28 (7.1)	1.13 [0.13; 10.04]	0.906	-
Pain intensity (NRS)	Baseline	17.47 ± 7.31	17.37 ± 6.49			
	9 weeks	14.19 ± 7.69	15.17 ± 6.78	-0.01 [-0.42; 0.41]	0.976	-0.14 [-0.65; 0.38]
	6 months	12.78 ± 7.69	14.17 ± 6.42	-0.13 [-0.60; 0.34]	0.574	-0.20 [-0.73; 0.34]
	12 months	11.60 ± 7.93	14.79 ± 7.28	-0.28 [-0.73; 0.16]	0.209	-0.42 [-0.98; 0.14]
Perceived improvement	9 weeks	3.37 ± 1.04	3.74 ± 1.46	-0.14 [-0.40; 0.11]	0.267	-0.29 [-0.80; 0.23]
	6 months	3.19 ± 1.49	3.77 ± 1.45	-0.20 [-0.46; 0.07]	0.143	-0.39 [-0.93; 0.14]
	12 months	3.08 ± 1.55	3.43 ± 1.37	-0.12 [-0.40; 0.16]	0.390	-0.24 [-0.79; 0.32]
ACT-based measures						
Activity engagement (CPAQ subscale)	Baseline	36.33 ± 10.22	37.37 ± 11.23			
	9 weeks	40.93 ± 13.60	37.69 ± 10.94	0.24 [-0.10; 0.58]	0.164	0.27 [-0.25; 0.78]
	6 months	43.67 ± 13.04	37.33 ± 13.02	0.48 [0.13; 0.83]	**0.008**	0.49 [-0.05; 1.03]
	12 months	45.52 ± 12.67	39.43 ± 8.93	0.52 [0.07; 0.96]	**0.023**	0.56 [-0.002; 1.12]
Pain willingness (CPAQ subscale)	Baseline	26.14 ± 10.01	26.24 ± 10.32			
	9 weeks	31.56 ± 11.91	29.77 ± 8.52	0.13 [-0.18; 0.44]	0.409	0.18 [-0.34; 0.69]
	6 months	33.07 ± 12.20	29.63 ± 9.38	0.28 [-0.05; 0.61]	0.095	0.32 [-0.22; 0.85]
	12 months	35.48 ± 11.59	28.71 ± 10.12	0.41 [0.02; 0.81]	**0.038**	0.62 [0.06; 1.19]
Chronic pain Acceptance (CPAQ total score)	Baseline	62.47 ± 17.77	63.61 ± 18.71			
	9 weeks	72.48 ± 23.20	67.46 ± 17.30	0.20 [-0.08; 0.49]	0.158	0.25 [-0.26; 0.76]
	6 months	76.74 ± 24.07	66.97 ± 19.33	0.42 [0.09; 0.76]	**0.014**	0.45 [-0.09; 0.99]
	12 months	81.00 ± 22.18	68.14 ± 17.97	0.50 [0.09; 0.90]	**0.017**	0.64 [0.07; 1.21]
Cognitive fusion (CFQ)	Baseline	23.35 ± 8.33	19.58 ± 9.47			
	9 weeks	18.96 ± 9.10	16.80 ± 8.13	-0.02 [-0.44; 0.41]	0.944	-0.25 [-0.77; 0.26]
	6 months	18.44 ± 9.62	18.57 ± 9.31	-0.34 [-0.81; 0.12]	0.144	-0.01 [-0.54; 0.52]
	12 months	16.92 ± 8.98	20.21 ± 8.73	-0.50 [-0.97; -0.03]	**0.038**	-0.37 [-0.93; 0.18]
Committed action (CAQ)	Baseline	68.74 ± 14.98	68.84 ± 16.87			
	9 weeks	72.63 ± 15.90	72.77 ± 14.36	0.02 [-0.33; 0.37]	0.910	0.01 [-0.50; 0.52]
	6 months	74.74 ± 16.12	71.30 ± 17.42	0.24 [-0.13; 0.60]	0.195	0.20 [-0.33; 0.74]
	12 months	77.72 ± 17.32	70.04 ± 17.85	0.27 [-0.19; 0.74]	0.240	0.44 [-0.12; 1.00]
Mental health-related outcomes						
Depressive symptom severity (QIDS-SR16)	Baseline	7.63 ± 3.80	7.89 ± 4.43			
	9 weeks	4.85 ± 3.29	6.29 ± 3.95	-0.27 [-0.65; 0.10]	0.148	-0.39 [-0.91; 0.13]
	6 months	5.15 ± 4.28	6.70 ± 4.47	-0.39 [-0.78; 0.01]	0.056	-0.35 [-0.89; 0.18]
	12 months	5.24 ± 4.27	6.07 ± 4.17	-0.14 [-0.56; 0.29]	0.516	-0.20 [-0.75; 0.36]

Onset of MDD (QIDS-SR16)	9 weeks	0 of 25 (0.0)	0 of 30 (0.0)	NA	NA	-
	6 months	0 of 24 (0.0)	2 of 25 (8.0)	NA	0.997	-
	12 months	0 of 22 (0.0)	1 of 24 (4.2)	NA	0.997	-
Onset of MDD (CIDI-SC)	9 weeks	0 of 24 (0.0)	0 of 26 (0.0)	NA	NA	-
	6 months	1 of 24 (4.2)	2 of 22 (9.1)	0.56 [0.03; 5.80]	0.631	-
	12 months	2 of 23 (8.7)	2 of 21 (9.5)	1.12 [0.13; 9.34]	0.910	-
Remission of MDD (QIDS-SR16)	9 weeks	1 of 2 (50.0)	2 of 5 (40.0)	0.65 [0.03; 6.77]	0.723	-
	6 months	0 of 3 (0.0)	3 of 5 (60.0)	NA	0.997	-
	12 months	1 of 3 (33.3)	3 of 4 (75.0)	0.37 [0.02; 2.92]	0.394	-
Remission of MDD (CIDI-SC)	9 weeks	0 of 3 (0.0)	0 of 9 (0.0)	NA	NA	-
	6 months	2 of 3 (66.7)	3 of 8 (37.5)	0.74 [0.10; 4.47]	0.742	-
	12 months	1 of 2 (50.0)	5 of 7 (71.4)	0.22 [0.01; 1.39]	0.172	-
Anxiety (GAD-7)	Baseline	7.16 ± 4.08	5.87 ± 4.08			
	9 weeks	4.00 ± 3.78	4.29 ± 3.37	-0.12 [-0.51; 0.28]	0.549	-0.08 [-0.59; 0.43]
	6 months	4.15 ± 3.67	4.50 ± 3.75	-0.29 [-0.76; 0.17]	0.212	-0.09 [-0.63; 0.44]
	12 months	3.64 ± 4.13	5.43 ± 4.16	-0.55 [-0.97; -0.12]	**0.013**	-0.43 [-0.99; 0.13]
Perceived stress (PSS)	Baseline	18.26 ± 6.60	17.76 ± 6.94			
	9 weeks	14.19 ± 8.70	13.86 ± 7.30	0.07 [-0.32; 0.46]	0.737	0.04 [-0.47; 0.55]
	6 months	14.93 ± 7.21	15.27 ± 9.05	-0.08 [-0.53; 0.37]	0.720	-0.04 [-0.57; 0.49]
	12 months	12.04 ± 7.60	16.89 ± 7.89	-0.49 [-0.91; -0.07]	**0.022**	-0.63 [-1.19; -0.06]
Insomnia (ISI)	Baseline	8.63 ± 4.91	9.08 ± 6.16			
	9 weeks	7.07 ± 5.05	6.80 ± 5.06	0.20 [-0.18; 0.58]	0.290	0.05 [-0.46; 0.57]
	6 months	6.81 ± 5.50	8.10 ± 5.74	-0.14 [-0.53; 0.25]	0.486	-0.23 [-0.76; 0.30]
	12 months	6.36 ± 6.53	7.57 ± 5.54	-0.06 [-0.45; 0.32]	0.744	-0.20 [-0.75; 0.35]
Alcohol consumption (AUDIT-10)	Baseline	2.58 ± 2.00	2.13 ± 1.79			
	9 weeks	2.15 ± 1.96	1.97 ± 1.77	-0.15 [-0.41; 0.12]	0.270	-0.10 [-0.61; 0.42]
	6 months	2.22 ± 1.50	1.60 ± 1.45	0.24 [-0.12; 0.60]	0.189	0.42 [-0.12; 0.96]
	12 months	1.60 ± 0.96	1.86 ± 1.46	-0.42 [-0.84; -0.003]	**0.048**	-0.21 [-0.76; 0.35]
Quality of life (AQoL-8D)	Baseline	69.44 ± 11.01	68.53 ± 10.26			
	9 weeks	72.58 ± 13.69	71.47 ± 11.38	-0.07 [-0.32; 0.19]	0.589	-0.09 [-0.60; 0.42]
	6 months	74.63 ± 12.17	70.97 ± 13.71	0.18 [-0.11; 0.47]	0.226	0.28 [-0.25; 0.82]
	12 months	79.35 ± 11.48	70.64 ± 10.81	0.49 [0.15; 0.83]	**0.006**	0.78 [0.21; 1.36]
Subjective prognosis of employment (SPE)	Baseline	1.51 ± 1.08	1.47 ± 0.98			
	9 weeks	1.22 ± 1.19	1.20 ± 1.05	0.18 [-0.18; 0.53]	0.329	0.02 [-0.49; 0.53]
	6 months	1.11 ± 1.01	1.13 ± 1.11	-0.06 [-0.49; 0.36]	0.763	-0.02 [-0.55; 0.51]
	12 months	1.00 ± 1.12	1.21 ± 1.10	-0.09 [-0.57; 0.39]	0.701	-0.19 [-0.75; 0.36]

Observed data were used for complete-case-analysis. Means ± SD or *n* (%) are reported for baseline, 9-weeks post-randomization, 6- and 12-month FU. [a]We report standardized regression estimates (β) with 95%-CI based on robust standard errors for between-group differences. Standardized regression estimates were adjusted for baseline except for analysis of primary outcome. Reliable change and deterioration is reported based on OR along with 95%-CI based on robust standard errors. Onset and remission of MDD is reported based on IRR along with 95%-CI based on robust standard errors. [b]*P* values are based on two-sided testing except for primary outcome, which was based on a one-sided test. Significant differences ($p \leq .05$) are printed in bold letters.

Table S3. Subjective ratings of the intervention, problems encountered and reasons for not starting or discontinuing the intervention at 9-weeks post-randomization (T1), 6-month (T2) and 12-month FU (T3).

	**9-weeks post (T1)**	**6-month FU (T2)**	**12-month FU (T3)**
	***N* (%)**	***N* (%)**	***N* (%)**
**Rating of the intervention content**			
Relevance of the training content (relevant or very relevant)	12 of 24 (50.0)	11 of 24 (45.8)	-
Usability of the intervention (good or very good)	-	18 of 24 (75.0)	-
Usability of the intervention platform (good or very good)	-	15 of 24 (62.5)	-
Satisfaction with eCoach support (satisfied or very satisfied)	-	16 of 24 (66.7)	-
Exclusive communication in written form with the eCoach	-	11 of 24 (45.8)	11 of 18 (61.1)
**Reasons for not beginning the intervention**			-
time constraints	1 of 4 (25.0)	1 of 3 (33.3)	-
technical problems	2 of 4 (50.0)	0 of 3 (0.0)	-
no motivation	0 of 4 (0.0)	1 of 3 (33.3)	-
not enough information	2 of 4 (50.0)	0 of 3 (0.0)	-
not enough support	1 of 4 (25.0)	0 of 3 (0.0)	-
**Problems with intervention conduct**			-
technical problems	6 of 10 (60.0)	8 of 10 (80.0)	-
problems like comprehension difficulties	5 of 10 (50.0)	0 of 10 (0.0)	-
difficulties to arrange calls with eCoach due to time restrictions	3 of 10 (30.0)	3 of 10 (30.0)	-
communication problems with eCoach	1 of 10 (10.0)	0 of 10 (0.0)	-
problems with internet connection	-	3 of 10 (30.0)	-
**Reasons for intervention dropout**			
intervention not useful	-	2 of 4 (50.0)	3 of 7 (42.9)
technical problems	-	1 of 4 (25.0)	2 of 7 (28.6)
no motivation	-	1 of 4 (25.0)	1 of 7 (14.3)
time constraints	-	1 of 4 (25.0)	1 of 7 (14.3)
high work load	-	0 of 4 (0.0)	1 of 7 (14.3)
illness	-	0 of 4 (0.0)	1 of 7 (14.3)
symptoms too severe	-	1 of 4 (25.0)	0 of 7 (0.0)

Ratings of the intervention content, reasons for not beginning the intervention, problems encountered and reasons for intervention dropout were based on item batteries with closed answering format formulated by the authors. Items were rated according to applicability in the corresponding subsamples at 9-weeks post-randomization (T1), 6-month FU (T2), 12-month FU (T3).

Table S4. Overview of negative effects of GET.ON Chronic Pain at 9-weeks post-randomization (T1) 6-month (T2) and 12-month FU (T3).

	**9-weeks post (T1), *N* (%)**		**6-month FU (T2), *N* (%)**		**12-month FU (T3), *N* (%)**	
	**Total**	**By intervention**	**Total**	**By intervention**	**Total**	**By intervention**
**Intrapersonal change**						
Improvement/worsening of symptoms	3 (6.0)	2 (6.5)	2 (5.7)	1 (5.3)	2 (4.5)	0 (0.0)
More/less trusting others	2 (4.0)	2 (6.5)	1 (2.9)	0 (0.0)	2 (4.5)	0 (0.0)
Severity of suffering from past experiences/events	3 (6.0)	2 (6.5)	2 (5.7)	2 (10.5)	2 (4.5)	2 (7.1)
Experiencing new thinking and behavior patterns as helpful/harmful	2 (4.0)	2 (6.5)	0 (0.0)	0 (0.0)	1 (2.3)	1 (3.6)
Feeling more/less lonely	1 (2.0)	1 (3.2)	0 (0.0)	0 (0.0)	0 (0.0)	0 (0.0)
Feeling dependent on IBI program	2 (4.0)	1 (3.2)	0 (0.0)	0 (0.0)	2 (4.5)	2 (7.1)
Difficulties to make decisions alone	1 (2.0)	1 (3.2)	2 (5.7)	0 (0.0)	1 (2.3)	1 (3.6)
Longer periods of feeling bad	4 (8.0)	2 (6.5)	5 (14.3)	1 (5.3)	5 (11.4)	2 (7.1)
As a human being changed to the negative	2 (4.0)	1 (3.2)	2 (5.7)	0 (0.0)	1 (2.3)	1 (3.6)
Thoughts/plans to commit suicide for the first time	0 (0.0)	0 (0.0)	0 (0.0)	0 (0.0)	0 (0.0)	0 (0.0)
**Relationship**						
More/less arguments/conflicts in relationship	6 (12.0)	3 (9.7)	2 (5.7)	2 (10.5)	4 (9.1)	3 (10.7)
Partner is jealous with therapeutic relationship	1 (2.0)	1 (3.2)	2 (5.7)	1 (5.3)	3 (6.8)	2 (7.1)
**Friends and Family**						
Worsened/improved relationship with family	2 (4.0)	1 (3.2)	2 (5.7)	1 (5.3)	3 (6.8)	2 (7.1)
Worsened/improved relationship with friends	0 (0.0)	0 (0.0)	1 (2.9)	1 (5.3)	1 (2.3)	1 (3.6)
**Stigma**						
Fear of others learning about the program usage	2 (4.0)	0 (0.0)	1 (2.9)	0 (0.0)	3 (6.8)	1 (3.6)
Worries about (potentially) increasing insurance fees	2 (4.0)	0 (0.0)	0 (0.0)	0 (0.0)	2 (4.5)	1 (3.6)
Financial worries	6 (12.0)	1 (3.2)	3 (8.6)	0 (0.0)	4 (9.1)	1 (3.6)
**Therapeutic malpractice by online-training /eCoach**						
Hurtful statements in online-training/by eCoach	0 (0.0)	0 (0.0)	0 (0.0)	0 (0.0)	0 (0.0)	0 (0.0)
Feeling of being made fun of by the online-training/by eCoach	0 (0.0)	0 (0.0)	0 (0.0)	0 (0.0)	0 (0.0)	0 (0.0)
Feeling of being forced to do exercises by the online-training/by eCoach	2 (4.0)	2 (6.5)	3 (8.6)	3 (15.8)	1 (2.3)	1 (3.6)
Feeling of data security not being ensured during the online-training	1 (2.0)	1 (3.2)	2 (5.7)	2 (10.5)	2 (4.5)	2 (7.1)
Neglect of hobbies and social contacts because of online-training	8 (16.0)	8 (25.8)	5 (14.3)	5 (26.3)	5 (11.4)	5 (17.9)
**Total score INEP (*N*)**	**50**	**31**	**35**	**19**	**44**	**28**

Summary of negative effects reported in intervention group at 9-weeks post-randomization (T1, n=27), 6-month FU (T2, n=27), 12-month FU (T3, n=25) based on the Inventory for the Assessment of Negative Effects of Psychotherapy (INEP). Negative effects attributed to the internet intervention GET.ON Chronic Pain are reported separately.
